# St. Thomas's Hospital: Presentation to Dr. Bristowe

**Published:** 1893-10-07

**Authors:** 


### ST. THOMAS'S HOSPITAL.

### PRESENTATION TO DR. BRISTOWE.

Tuesday, the 3rd inst., was a memorable day in the memory of this institution. Under the presidency of Mr. J. G. Wainwright, the Treasurer, and in the presence of Lord Thring (who delivered an excellent address to the students), the Lord Mayor, Sir Edwin Saunders, the members of the consulting and acting medical staff, and a crowded audience of governors, students, and friends, Dr. Bristowe, F.R.S., Consulting Physician, was presented with an oil portrait of himself, together with an address and album containing the names of upwards of 400 subscribers, being his colleagues and old pupils. The portrait, which was hung in this year's Academy, proved an excellent likeness, and the interest attaching to it was increased by the fact that it had been painted by Miss Bristowe, the daughter of the recipient. Dr. Ord, the Senior Physician, made the presentation in an eloquent and powerful address. He enumerated the many and great services which Dr. Bristowe had rendered to St. Thomas's Hospital during the last forty years; dwelt with eloquence upon the value of the public work of Dr. Bristowe, and especially of his book upon medicine; spoke of him as a well-beloved colleague and devoted friend; and declared that in years to come posterity would gratefully recognise the importance of the services which Dr. Bristowe had rendered to the community during a long life—services which he hoped would receive early and lasting recognition from the world, who had too long ignored them. The kindly sympathy and affectionate interest which Dr. Bristowe invariably took in the advancement of students had endeared him to many generations of St. Thomas's men, who were grateful to have the present opportunity of showing their gratitude, however inadequately, and to testify by their presence the warmth of the feelings which animated them towards their distinguished Consulting Physician. Dr. Ord's address was frequently applauded, and at its conclusion Dr. Bristowe, who was greatly moved, in a few well-chosen words, expressed his appreciation of the honour and his thanks for the kindness which had been shown to him.

The following students are the medallists of the year:—E. A. Saunders, Balham, the Mead medal in practical medicine; S. W. F. Richardson, the Cheselden medal in surgery and surgical anatomy; and S. W. F. Richardson, the treasurer's gold medal for general proficiency and good conduct. Mr. G. H. Makins, dean of the hospital, stated that, compared with other schools, the general average of St. Thomas's School had been good.

The Governors' Hall was crowded, and many people were unable to obtain admission. The whole proceedings went off with much *éclat*. We congratulate Dr. Bristowe upon this remarkable and appreciative demonstration of the affectionate recognition which his devoted services have evoked, and which cannot fail to still further extend the influence and reputation of the great medical school with which he was so long identified.

